# Cystitis in Women

**Published:** 1893-04

**Authors:** 


### CYSTITIS IN WOMEN.

In a discussion of a paper by Dr. Amelia J. Prior on this subject in the Cincinnati Medical Society, Dr. Stanton said (*Lancet-Clinic*, March 25): I think we all agree that local treatment is the most important in these cases. I do not altogether agree with the essayist in reference to constitutional treatment. It seems to me that palliation can often be secured by constitutional treatment. For instancs, an irritation in the bladder, produced by the salts of the urine, will be relieved by remedies which increase the flow of the water, thus diluting the urine. Thus belladonna with diuretics will frequently give relief. Couchgrass is frequently prescribed with great benefit in these cases. I believe the greatest benefit is secured by the increased flow of water, causing a diminution of the irritation produced by the salts yf the urine or whatever irritating substance may be present. The principal good is obtained from the administration of soothing remedies, which relieve the irritation of the bladder, especially by causing dilution of the salts of the urine. I think the local treatment, while it is of the most importance, certainly is not the only treatment, but is an excellent palliative measure. With regard to the use of local remedies in this disease. I have not had much experience with boracic acid, but where I have used it I have found it beneficial. I certainly would give preference to it in a recent case in which other remedies had not been tried, but otherwise I would resort to the use of nitrate of silver in a weak solution, gr. ½ to the ounce, injecting the fluid into the bladder and withdrawing it by means of a syringe soon after the injection. Years ago Dr. Shaw read a paper in which he mentioned having used a solution of grs. xl to the ounce. He certainly had a very happy result, but I should hesitate to use an injection of the strength. Dilatation of the urethra and frequent evacuation of the bladder will give a great deal of relief and assist in the cure of these cases. Distention of the bladder aggravates the disease, and relief should be given to the organ, and it should not be permitted to be distended. Cases of pericystitis are sometimes quite annoying where there is retention of urine after delivery, and the catheter has to be used. Dilatation sometimes would be likely to give relief, and I think it is an operation which is not resorted to as often as it ought to be in a disease aitended with as much distress as this. There are new methods of treatment recommended for cystitis. One is curetting the bladder, and another is dilating and swabbing out the bladder. These I have never tried, and it seems to me both are methods more likely to be productive of evil results than good, unless performed by an experinced operator.—*Maryland Med. Journal.*

